# Уровень продукции активных форм кислорода в моноцитах периферической крови у пациентов с болезнью Грейвса в динамике после радионуклидного лечения

**DOI:** 10.14341/probl13444

**Published:** 2025-05-20

**Authors:** Д. В. Фомина, С. А. Догадин, А. А. Савченко, И. И. Гвоздев

**Affiliations:** Красноярский государственный медицинский университет им. профессора В.Ф. Войно-Ясенецкого; Красноярский государственный медицинский университет им. профессора В.Ф. Войно-Ясенецкого; Краевая клиническая больница; Красноярский государственный медицинский университет им. профессора В.Ф. Войно-Ясенецкого; Красноярский научный центр Сибирского отделения Российской академии наук; Красноярский научный центр Сибирского отделения Российской академии наук

**Keywords:** болезнь Грейвса, моноциты, АФК, активные формы кислорода, хемилюминесценция, радиойодтерапия, РЙТ

## Abstract

**ОБОСНОВАНИЕ:**

ОБОСНОВАНИЕ. Аутоиммунные заболевания, такие как болезнь Грейвса, являются сложными патологиями, часто требующими интенсивного и длительного лечения. Однако механизмы, способствующие развитию и поддержанию этой патологии, до сих пор остаются не до конца изученными. Понимание роли врожденного иммунного ответа, особенно в контексте реакций моноцитов, в развитии болезни Грейвса остается малоизученным аспектом.

**ЦЕЛЬ:**

ЦЕЛЬ. Изучить уровень продукции активных форм кислорода (АФК) в моноцитах у пациентов с болезнью Грейвса после радиойодтерапии (РЙТ) для выявления уровня активации макрофагально-моноцитарной системы.

**МАТЕРИАЛЫ И МЕТОДЫ:**

МАТЕРИАЛЫ И МЕТОДЫ. В исследование включено 48 пациенток с болезнью Грейвса возрастом от 18 до 65 лет. Гормональный статус и активность моноцитов анализировали до и через 1, 3 и 6 месяцев после радионуклидного лечения. Уровень АФК определяли с помощью хемилюминесценции спонтанной и индуцированной люминолом и люцегинином.

**РЕЗУЛЬТАТЫ:**

РЕЗУЛЬТАТЫ. У пациентов с болезнью Грейвса выявлено снижение уровня синтеза АФК в моноцитах по сравнению с контрольной группой. Эти изменения наблюдались как до, так и после радионуклидного лечения. Показано, что изменения в производстве АФК не зависят от тиреоидной функции и уровня аутоантител. Снижение продукции также отмечено в отношении вторичных АФК в моноцитах.

**ЗАКЛЮЧЕНИЕ:**

ЗАКЛЮЧЕНИЕ. Изменения свидетельствуют о потенциальном иммуносупрессивном воздействии радионуклидного лечения и его влиянии на активность НАДФН-оксидазы моноцитов. Низкий уровень синтеза АФК коррелирует с ингибированием аутоиммунного процесса и снижением активации макрофагально-моноцитарной системы. Исследование подтверждает важность роли моноцитов в системе продукции АФК и их влияние на аутоиммунный процесс при болезни Грейвса. Эти результаты могут иметь клиническую значимость и способствовать разработке новых иммунотропных стратегий для лечения данного заболевания.

## Abstract (en)

**BACKGROUND:**

BACKGROUND: Autoimmune diseases such as Graves’ disease are complex pathologies that often require intensive and long-term treatment. However, the mechanisms contributing to the development and maintenance of this pathology are still not fully understood. Understanding the role of the innate immune response, especially in the context of monocytes, in the development of Graves’ disease remains a poorly understood aspect.

**AIM:**

AIM: To study the level of ROS production in monocytes in patients with Graves’ disease after RAI to identify the level of activation of the macrophage-monocyte system.

**MATERIALS AND METHODS:**

MATERIALS AND METHODS: The hormonal status and monocyte activity were analyzed before and 1, 3 and 6 months after RAI. The level of ROS was determined using spontaneous and luminol- and luceginin-derived chemiluminescence.

**RESULTS:**

RESULTS: The study included 48 patients with Graves’ disease, aged from 18 to 65 years. In patients with Graves’ disease, a decrease in the intensity of free radical processes in monocytes was detected compared to the control group. These changes were observed both before and after RAI. It has been shown that changes in ROS production are independent of thyroid function and antibody levels. The changes indicate the potential immunosuppressive effects of radionuclide treatment and its effect on monocyte NADPH oxidase activity. A decrease in the production of secondary ROS in monocytes was also noted, which may indicate a decrease in the activation of monocytes during antigenic stimulation and inhibition of autoimmune processes.

**CONCLUSION:**

CONCLUSION: Reduced metabolic activity of monocytes and low levels of ROS synthesis correlate with inhibition of the autoimmune process and decreased activation of the macrophage-monocyte system. The study confirms the importance of the role of monocytes in the ROS production system and their influence on the autoimmune process in Graves’ disease. These results may have clinical significance and contribute to the development of new immunotropic strategies for the treatment of this disease.

## ОБОСНОВАНИЕ

Болезнь Грейвса представляет собой органоспецифическое аутоиммунное заболевание, характеризующееся наличием антител к рецепторам тиреотропного гормона (АТ-рТТГ) [1]. АТ-рТТГ могут связываться с тиреотропным рецептором, экспрессируемым на фолликулярных клетках щитовидной железы (ЩЖ), и стимулировать фолликулярные клетки ЩЖ к продукции и высвобождению избыточного количества тиреоидных гормонов [2]. Болезнь Грейвса является многофакторным заболеванием, вызванным взаимодействием между генами предрасположенности, факторами окружающей среды и иммунитетом [1].

Многие исследования подтверждают наличие активированных Т- и В-клеток, как на периферии, так и в ЩЖ у пациентов с болезнью Грейвса [1–4]. В отличие от четко определенной роли Т и B-клеток в иммунопатогенезе болезни Грейвса, мало что известно о роли моноцитов. Моноциты относятся к ключевым компонентам врожденной иммунной системы [1]. Моноциты и тканевые макрофаги являются ведущими клетками иммунного ответа организма, обеспечивая развитие местного и системного воcпаления, процессинг антигенов и их презентацию [5]. Представляя антигены Т-клеткам и секретируя воспалительные цитокины, такие как фактор некроза опухоли-α (ФНО-α), интерлейкин-2 (ИЛ-2), ИЛ-4, ИЛ-6, фактор активации В-клеток (BAFF), моноциты являются регуляторами, которые способствуют активации клеток адаптивного иммунитета [5]. В процессе реализации функциональной активности (включая, фагоцитоз) моноциты синтезируют широкий спектр активных форм кислорода (АФК). АФК участвуют во многих клеточных процессах, таких как фагоцитоз; синтез гормонов и передача сигналов; активация рецепторов, связанных с G-белком, киназ/фосфатаз и факторов транскрипции; и экспрессии генов. Однако в патофизиологических условиях АФК также стимулируют воспаление и фиброз [6].

Известно, что повышенная продукция АФК играет существенную роль в инициации и персистенции аутоиммунного процесса при болезни Грейвса, так как уровень генерации АФК тесно связан с активацией антигенспецифических Th-клеток и продукцией антител В-лимфоцитами [7]. Влияние радионуклидного лечения (РЙТ) на состояние макрофагально-моноцитарной системы при болезни Грейвса до сих пор не изучено. Однако процессы киллинга в моноцитах обеспечиваются при участии кислородозависимых систем, которые представлены миелопероксидазой, продуктами респираторного взрыва, возникающего при активации фагоцитирующих клеток: супероксидным анион-радикалом, гидроксильным радикалом, галогенами, перекисью водорода, синглетным кислородом [8]. В связи с этим оценка уровня генерации АФК в моноцитах представляется важной количественной и качественной характеристикой, не только отражающей функциональную активность моноцитов при болезни Грейвса, но и определяющей участие клеток макрофагально-моноцитарной системы в механизмах повторной иммунной атаки и рецидивировании заболевания.

## ЦЕЛЬ ИССЛЕДОВАНИЯ

Изучить уровень продукции АФК в моноцитах периферической крови у пациентов с болезнью Грейвса в динамике радионуклидного лечения для выявления уровня активации макрофагально-моноцитарной системы и ее роли в персистенции аутоиммунного процесса.

## МАТЕРИАЛЫ И МЕТОДЫ

## Место и время проведения исследования

Место проведения. Исследование выполнено на базе эндокринологического центра КГБУЗ «Краевая клиническая больница», г. Красноярск.

Время исследования. Наблюдение пациентов осуществлялось с января 2019-го по май 2021 гг.

## Изучаемые популяции

Две популяции: пациенты с болезнью Грейвса и группа контроля — здоровые женщины.

Популяция «пациенты с болезнью Грейвса»

Критерии включения: женщины в возрасте от 18 до 55 лет с верифицированным диагнозом «Болезнь Грейвса», которым планируется проведение радикального лечения методом радиойодтерапии.

Популяция «контроль»

Критерии включения: практически здоровые женщины возрастом от 18 до 55 лет, без отягощенного анамнеза по заболеваниям щитовидной железы (ЩЖ) у себя и кровных родственников, а также с отсутствием структурных изменений ЩЖ при ультразвуковом исследовании (УЗИ).

Критерии исключения: узловой/многоузловой токсический зоб; беременность и лактация, острые психические заболевания, злокачественные новообразования, другие эндокринопатии, обострение хронических заболеваний, острые заболевания, болезни крови, инфекционные и аутоиммунные заболевания, прием йодсодержащих препаратов.

## Способ формирования выборки из изучаемой популяции (или нескольких выборок из нескольких изучаемых популяций)

Формирование группы пациентов с болезнью Грейвса проводилось сплошным способом подбора.

## Дизайн исследования

Контролируемое, одноцентровое, открытое, проспективное, когортное исследование.

## Методы

Диагноз подтверждали на основании специфических жалоб, данных гормонального исследования и повышения уровня АТ-рТТГ согласно клиническим рекомендациям Российской ассоциации эндокринологов [8].

Для определения уровня гормонов щитовидной железы: ТТГ и свободного тироксина (свТ4) использовали метод иммунохемилюминесцентного анализа (ИХЛА) на анализаторе Architecti1000sr (Abbott Diagnostics, США), референсные значения: 0,35–4,94 мМЕ/л и 9,01–19,05 пмоль/л соответственно. Определение уровня свободного трийодтиронина (свТ3) проводили методом ИФА с использованием тест-систем «ДС-ИФА-ТИРОИД-Т3 свободный» (ООО «НПО «Диагностические системы»), референсный интервал 2,0–5,7 пмоль/л. Уровень АТ-рТТГ оценивался методом ИФА при помощи набора Medizym T.R.A. (MEDIPAN GmbH, Германия), референсный уровень 0,0–1,0 МЕд/л, серая зона >1,5 МЕд/л. УЗИ щитовидной железы выполнено на аппарате iU22 (Philips, США). Расчет объема ЩЖ проводился по формуле, предложенной J. Brunn (1981 г.).

Для измерения клеточных АФК использовали взвесь моноцитов (2 млн/мл) в сбалансированном солевом растворе Хенкса 240 мкл («ПанЭко», Россия) с добавлением 20 мкл донорской сыворотки AB(IV) Rh(–), в качестве индикатора добавляли люцигенин или люминол 50 мкл («Sigma», США) в концентрации 10⁻⁵ М. Для определения индуцированной люминесценции использовали 40 мкл опсонизированного зимозана (зимозан-индуцированная хемилюминесценция), объем раствора Хенкса составил 200 мкл [14]. При использовании люминола определялся общий пул АФК, с помощью люцегинина оценивался пул АФК, образуемый НАДФН-оксидазой. Активность и кинетику хемилюминесцентной реакции количественно определяли с помощью люминометра БЛМ-3607 (ООО «МедБиоТех», Красноярск). Измерение интенсивности свечения осуществляли в течение 90 мин на 36-канальном анализаторе. Определяли следующие характеристики хемилюминесцентной кривой: время выхода на максимум (Тmax), характеризующее скорость развития реакции, максимальная интенсивность свечения (Imax), которая отражает максимальный уровень синтеза АФК, а также площадь под кривой (S), характеризующую суммарный синтез АФК за 90 мин исследования. Индекс активации оценивали по отношению площади индуцированной хемилюминесценции к площади спонтанной.

## Статистический анализ

Для оценки соответствия распределения количественных данных закону нормального распределения использовался тест Шапиро-Уилка. При нормальном распределении показателей описание выборки производили с помощью медианы (Ме) и интерквартильного размаха (Q1–Q3). При сравнении двух независимых выборок достоверность различий оценивали по непараметрическому критерию Манна-Уитни (Mann-Whitney U test), для зависимых выборок использовался критерий Вилкоксона. Различия считали статистически значимыми при уровне p<0,05. Обработку полученных данных выполняли с использованием пакета программ Statistica 8.0 (StatSoftInc., 2007).

## Этическая экспертиза

Протокол исследования одобрен локальным этическим комитетом КГБУЗ «Краевая клиническая больница» № 162/1 от 21.03.2019 г.

## РЕЗУЛЬТАТЫ

В исследование было включено 48 пациенток с подтвержденным диагнозом «Болезнь Грейвса» в возрасте от 18 до 55 лет (средний возраст составил 46,42±15,26 года). Пациенты, включенные в исследование, ранее находились под наблюдением лечащего врача-эндокринолога и получали медикаментозное лечение тиреостатиками. Среди пациентов не выявлено курящих на момент включения в исследование. Пациентам, курившим на момент постановки диагноза, был рекомендован отказ от курения; как известно, курение потенцирует процессы оксидативного стресса и играет роль в усугублении аутоиммунного процесса при болезни Грейвса, а также способствует рецидиву заболевания после РЙТ. Среди участников исследования также не было пациентов с эндокринной офтальмопатией. Учитывая данные о том, что радиотерапия может ухудшать состояние офтальмопатии, наличие такого состояния рассматривалось как фактор в пользу хирургического вмешательства при выборе метода радикального лечения.

Гормональный статус у пациентов с болезнью Грейвса исследовали исходно — в день проведения РЙТ и через 1, 3 и 6 мес после радионуклидного лечения (табл. 1).

**Table:** Таблица 1. Гормональная характеристика пациентов с болезнью Грейвса в динамике

	Визит	
Перед РЙТ	Через 1 месяц	Через 3 месяца	Через 6 месяцев
1	2	3	4
ТТГ, мЕд/л	0,08 (0,01; 0,45)	2,38 (0,92; 5,71)	2,36 (0,68; 7,80)	2,13 (0,99; 3,44)	p1–2<0,001 p1–3<0,001 p1–4<0,001
свТ4, пмоль/л	17,49 (12,88; 21,44)	13,32 (10,19; 18,05)	12,31 (9,56; 14,60)	13,23 (12,02; 15,78)	p1–2=0,015 p1–3<0,001 p1–4<0,001 p2–4=0,049
свТ3, пмоль/л	5,28 (4,32; 7,38)	2,53 (1,68; 3,88)	3,20 (2,20; 4,21)	3,30 (2,50; 4,44)	p1–2<0,001 p1–3<0,001 p1–4<0,001 p2–4=0,044
АТ к рТТГ, МЕд/л	12,80 (5,77; 26,58)	1,66 (0,42; 4,50)	2,36 (1,32; 3,70)	1,48 (0,86; 2,91)	p1–2<0,001 p1–3<0,001 p1–4<0,001

Перед проведением РЙТ у пациентов был зафиксирован субклинический тиреотоксикоз, возникший после отмены тиреостатиков в рамках подготовки к лечению. При дальнейшем наблюдении за пациентами выявляется нормализация уровня ТТГ, снижение свободных фракций тиреоидных гормонов. При развитии послерадиационного гипотиреоза назначалась заместительная терапия левотироксином натрия для поддержания эутиреоидного состояния. Уровень АТ-рТТГ снизился после проведения РЙТ.

Для определения уровня продукции АФК в моноцитах периферической крови у пациентов с болезнью Грейвса проводили исследование с помощью хемилюминесцентного метода (табл. 2 и 3).

**Table:** Таблица 2. Показатели люцигенин-зависимой хемилюминесценции моноцитов крови пациентов с болезнью Грейвса в динамике, Ме (Q1– Q3)

Параметр	Контроль (n=23)	Перед РЙТ	Через 1 месяц после РЙТ	Через 3 месяца после РЙТ	Через 6 месяцев после РЙТ	
1	2	3	4	5
Люцигенин-зависимая спонтанная хемилюминесценция
Tmax, c	2039,5 (1288,5; 2260)	971 (443; 1578)	402 (303,5–655,5)	426 (266–1765)	1116 (256–1666)	p1-2<0,001 p1-3<0,001 p1-4=0,004 p1-5=0,036
Imax, отн.ед	394 (147,5; 573,5)	108 (65; 569)	66 (56,5–100,5)	145 (97–268)	94 (68–145)	p1-2=0,038 p1-3=0,001 p1-4=0,001 p1-5=0,001
S, отн.ед.×10^5	14,8 (5,29; 24,81)	3,60 (2,15; 12,72)	1,87 (0,71–2,71)	3,72 (2,13–8,79)	2,8 (2,15–4,05)	p1-2=0,011 p1-3=0,001 p1-4=0,001 p1-5=0,001
Люцигенин-зависимая зимозан-индуцированная хемилюминесценция
Tmax, c	1862 (1506; 2231,5)	1661 (1083; 2128)	1208,5 (1059–1420)	1942,5 (638,5–3392)	1425 (1271–1643)	p1-3=0,011
Imax, отн.ед.	947 (313; 1986,5)	231 (133; 685)	65 (59–70)	380,5 (134,5–4243,5)	290 (139–380)	p1-2=0,005 p1-3=0,005 p1-4=0,018 p1-5=0,012
S, отн.ед.×10^5	34,75 (10,56; 77,15)	7,24 (5,36; 18,27)	1,40 (1,24–2,22)	12,35 (5,12–144,16)	10,47 (5,73–11,83)	p1-2=0,003 p1-3=0,002 p1-4=0,011 p1-5=0,007
Индекс активации	2,81 (1,38; 7,51)	1,98 (1,39; 3,67)	1,55 (0,48–2,71)	1,04 (0,8–3,19)	1,41 (1,05–4,53)	

**Table:** Таблица 3. Показатели люминол-зависимой хемилюминесценции моноцитов крови пациентов с болезнью Грейвса в динамике, Ме (Q1–Q3)

Параметр	Контроль (n=23)	Перед РЙТ	Через 1 месяц после РЙТ	Через 3 месяца после РЙТ	Через 6 месяцев после РЙТ	
1	2	3	4	5
Люминол-зависимая спонтанная хемилюминесценция	
Tmax, c	1753 (399; 2766)	1059 (279; 1908)	1072,5 (82–2225)	479 (345–1341)	477,5 (360–1753)	p1-4=0,046
Imax, отн.ед	933 (497; 2251)	769,5 (151; 1498)	165,5 (53,5–471,5)	741 (145–1430)	642 (86–1209)	p1-3=0,008
S, отн.ед.×10^5	23,78 (13,44; 80,3)	18,87 (3,89; 44,55)	5,22 (1,38–12,78)	15,49 (5,71–21,42)	14,85 (2,72–21,42)	p1-3=0,009 p1-4=0,018 p1-5=0,033
Люминол-зависимая зимозан-индуцированная хемилюминесценция	
Tmax, c	1506 (902; 1971)	1285 (369; 2080)	2063 (1428–2614)	2684 (663–4734,5)	1031,5 (316–2330)	
Imax, отн.ед.	2284 (1080; 7136)	3121 (416; 5692)	234 (55–731)	1590 (857–4100)	1012,5 (87–2120)	p1-3=0,005 p1-4=0,022 p1-5=0,019 p 2-5=0,017
S, отн.ед.×10^5	80,18 (28,77; 311,06)	91,26 (11,57; 140,8)	8,09 (1,97–23,63)	38,8 (15,28–143,68)	26,48 (3,13–7,86)	p1-3=0,008 p1-4=0,017 p1-5=0,019 p 2-5=0,017
Индекс активации	3,31 (1,65; 4,77)	3,04 (1,83; 4,18)	2,3 (1,11–8,72)	1,37 (0,81–1,96)	1,59 (0,88–3,15)	p1-4=0,020 p 2-5=0,017

Выявлено снижение всех показателей люцигенин-зависимой спонтанной хемилюминесценции у пациентов с болезнью Грейвса по сравнению с контрольной группой: снижена скорость протекания реакции и время выхода на максимум (Т), максимальный уровень свечения (Imax) и, соответственно, суммарный пул АФК (S). Различия определены для всех точек наблюдения за пациентами, в том числе у пациентов через 6 мес после терапии радиоактивным йодом.

В ходе зимозан-индуцированной реакции значимые различия по скорости реакции установлены при сравнении контрольной группы и пациентов спустя 1 мес после РЙТ. Максимум интенсивности (Imax) и общее количество АФК (S) у пациентов с болезнью Грейвса во всех точках наблюдения ниже, чем контрольные показатели.

Не выявлено статистически значимых различий между контрольной группой и пациентами с болезнью Грейвса до РЙТ по параметрам люминол-зависимой хемилюминесценции моноцитов.

После проведенной РЙТ отмечено статистически значимое снижение показателей при сравнении контрольной группы и пациентов: во всех точках наблюдения при люминол-зависимой спонтанной ХЛ снижена S, а при ХЛ, индуцированной зимозаном, как интенсивность реакции, так и общий пул АФК значительной снижены.

Интенсивность спонтанной реакции отличалась от контрольного уровня только у пациентов через 1 мес после РЙТ. Скорость развития спонтанной реакции (Т) и индекс активации отличался при сравнении контроля и пациентов через 3 мес после РЙТ.

При анализе динамики показателей пациентов до и через 6 мес в ходе лечения выявлено статистически значимое снижение максимального уровня свечения (Imax) и, соответственно, общего числа АФК (S), а также снижение индекса активации люминол-зависимой зимозан-индуцированной хемилюминесценции.

## ОБСУЖДЕНИЕ

Моноциты являются важными клетками врожденной иммунной системы и играют специфическую роль в контроле, развитии и эскалации иммунологических процессов, осуществляя фагоцитоз, презентируя антигены и секретируя цитокины.

В ходе нашего исследования выявлено, что интенсивность, время развития хемилюминесцентной реакции и суммарный уровень продукции первичных АФК в моноцитах периферической крови у пациентов с болезнью Грейвса, по сравнению с показателями контрольной группы, снижены, как до радионуклидного лечения, так и при обследовании в динамике после РЙТ. Причем, если снижение генерации пула первичных АФК у пациентов с болезнью Грейвса до РЙТ может отражать долгосрочные иммуномодулирующие влияния консервативной терапии тиамазолом, то низкая интенсивность кислородного метаболизма в динамике после радионуклидного лечения, вероятно, связана с иммуносупрессивным воздействием ¹³¹I на активность НАДФН-оксидазы моноцитов. При этом при антигенном воздействии на моноциты периферической крови пациентов с болезнью Грейвса также наблюдалось закономерное снижение максимального уровня и суммарного синтеза АФК при отсутствии изменения скорости образования в системе первичных АФК. Однако транзиторное повышение скорости, максимального уровня и суммарной продукции первичных АФК, как исходно, так и в стимулированных моноцитах крови у пациентов с болезнью Грейвса, через 3 месяца, в сравнении с соответствующими показателями продукции АФК, выявляемыми через 1 месяц после радионуклидного лечения, свидетельствует о развитии компенсаторной реакции, связанной с некоторой интенсификацией генерации свободно-радикальных процессов и удалением избыточного пула первичных АФК для поддержания нормального функционирования НАДФН-оксидазного комплекса. Похожая закономерность у пациентов с болезнью Грейвса через 3 месяца после радионуклидного лечения наблюдалась и в уровне продукции вторичных АФК в моноцитах периферической крови, но при снижении исходной скорости их образования, до стимуляции моноцитов. Следует отметить, что снижение индекса активации моноцитов в системе продукции вторичных АФК свидетельствует о снижении активации при антигенной стимуляции моноцитов и отражает ингибирование аутоиммунного процесса.

Повышенный уровень продукции АФК при болезни Грейвса может являться пусковым механизмом не только в индукции гиперметаболического состояния, но и усугублять дефект регуляторных Т-клеток, способствуя развитию иммунной атаки и рецидиву заболевания [9]. Предыдущие исследования показали, что у пациентов с дебютом болезни Грейвса при манифестации гипертиреоза увеличена интенсивность образования вторичных АФК в нейтрофилах крови, как в состоянии покоя, так и при антигенной стимуляции клеток иммунной системы, но при соответствии скорости и суммарной продукции вторичных АФК контрольному диапазону [10]. Однако выявленный в настоящем исследовании низкий уровень активации кислород-зависимого метаболизма моноцитов наблюдался как на фоне субклинического гипертиреоза, до приема терапевтической активности 131I, так и в динамике после радионуклидного лечения, на фоне компенсированного пострадиационного гипотиреоза. Следовательно, у пациентов с болезнью Грейвса уровень продукции первичных и вторичных АФК в моноцитах периферической крови не зависит от функции ЩЖ и титра АТ-рТТГ, и может являться самостоятельным триггерным механизмом активации аутоиммунного процесса. В то же время у пациентов с болезнью Грейвса индукция эутиреоидного состояния и модуляция иммунного ответа на фоне консервативной терапии тиамазолом, а также последующее воздействие 131I, вероятно, оказывает иммуносупрессивное действие на систему иммунитета.

Можно заключить, что у пациентов с болезнью Грейвса, как до, так и в динамике после радионуклидного лечения, понижение интенсивности респираторного взрыва моноцитов определяется низким фоновым и индуцированным синтезом первичных и вторичных АФК в моноцитах крови, независимо от тиреоидной функции и снижения титра АТ-рТТГ, что может являться не только проявлением ингибирования антителозависимой цитотоксической активности моноцитов, но и индикатором реактивации аутоиммунного процесса при развитии рецидива заболевания.

## Сопоставление с другими публикациями

При многих аутоиммунных заболеваниях наличие аутоантител и аутореактивных Т- и B-клеток указывает на то, что адаптивная иммунная система играет решающее значение для патогенеза, но это не может полностью объяснить развитие аутоиммунных заболеваний, и врожденный иммунный ответ может играть важную и решающую роль в их патогенезе [11].

Изменение количества моноцитов является отличительной чертой многих аутоиммунных заболеваний. Относительно патологической роли моноцитов в развитии болезни Грейвса мало что известно. По данным литературы, описано значительное увеличение количества моноцитов у пациентов с болезнью Грейвса. Некоторые авторы предполагают патологическую роль моноцитов в продукции АТ-рТТГ [12].

В исследовании Alvaro González с соавт. при фенотипировании мононуклеарных клеток периферической крови показано, что у пациентов с болезнью Грейвса на разных стадиях заболевания моноциты находились в состоянии активации и дифференцировки более выраженном, чем у здоровых лиц [13]. Помимо высокой доли активированных подмножеств моноцитов, также выявляется наличие индуцибельной синтазы оксида азота (iNOS) в моноцитах пациентов с болезнью Грейвса, данный факт указывает на то, что выработка NO зависит от состояния созревания моноцитов [14]. Индуцируемая изоформа iNOS обычно отсутствует в физиологических условиях. Его экспрессия индуцируется в патологических ситуациях [15].

В дополнение к анализу уровня продукции активных форм кислорода (АФК) в моноцитах можно рассмотреть исследование других маркеров оксидативного стресса для предикции течения заболевания на фоне радионуклидного лечения. Одним из таких маркеров является малоновый диальдегид (MDA), который образуется при перекисном окислении липидов. MDA является индикатором уровня липидной пероксидации и может быть использован для оценки степени оксидативного стресса у пациентов с болезнью Грейвса, что может предоставить дополнительную информацию о патогенезе и прогнозе заболевания.

## Клиническая значимость результатов

Выявленные изменения продукции первичных АФК в моноцитах периферической крови у пациентов с болезнью Грейвса свидетельствуют о модуляции адаптивной иммунной системы моноцитами. Несмотря на то, что клиническая интерпретация этих данных для разработки этиотропной иммунной терапии остается сложной задачей, наше исследование подтверждает важность учета роли моноцитов в разработке терапевтической стратегии при аутоиммунных заболеваниях.

## Ограничения исследования

При интерпретации данных следует учитывать, что моноциты представляют собой гетерогенную популяцию с фенотипическими и функциональными различиями. При заболевании рецидивирующего характера, таком как болезнь Грейвса, следует ожидать, что имеет место динамический процесс элиминации стимулированных субпопуляций и регенерации моноцитов, приводящий к эффекту обратной связи [16].

## ЗАКЛЮЧЕНИЕ

Таким образом, у пациентов с болезнью Грейвса в динамике после радионуклидного лечения установлены однонаправленные изменения в системе продукции первичных и вторичных АФК моноцитов. Пониженная активность НАДФН-оксидазы и низкий уровень интенсивности образования вторичных АФК напрямую связаны с ингибированием аутоиммунного процесса и снижением уровня активации макрофагально-моноцитарной системы. Вместе с тем отсутствие связи изменений уровня продукции АФК моноцитами с изменениями показателей тиреоидного статуса и титра АТ-рТТГ у пациентов с болезнью Грейвса, как до, так и после радионуклидного лечения, раскрывает свободно-радикальный механизм персистенции аутоиммунного процесса, который может быть применен для разработки иммунотропной терапии заболевания.

## ДОПОЛНИТЕЛЬНАЯ ИНФОРМАЦИЯ

Источники финансирования. Исследование выполнялось на базе лаборатории молекулярно-клеточной физиологии и патологии Федерального исследовательского центра «Красноярский научный центр Сибирского отделения Российской академии наук», обособленное подразделение «НИИ медицинских проблем Севера».

Конфликт интересов. Авторы декларируют отсутствие явных и потенциальных конфликтов интересов, связанных с содержанием настоящей статьи.

Участие авторов. Фомина Д.В. — получение, анализ данных, интерпретация результатов, написание статьи; Савченко А.А. — концепция и дизайн исследования, внесение в рукопись существенной (важной) правки с целью повышения научной ценности статьи; Догадин С.А. — концепция и дизайн исследования, внесение в рукопись существенной (важной) правки с целью повышения научной ценности статьи; Гвоздев И.И. — получение и интерпретация результатов. Все авторы одобрили финальную версию статьи перед публикацией, выразили согласие нести ответственность за все аспекты работы, подразумевающую надлежащее изучение и решение вопросов, связанных с точностью или добросовестностью любой части работы.
